# A framework for facilitating sustainable One Health collaboration across sectors at the national level in the European Union/European Economic Area

**DOI:** 10.2807/1560-7917.ES.2025.30.43.2500218

**Published:** 2025-10-30

**Authors:** Juliette Hoefle-Bénard, Carmen Varela Santos, Ole Heuer, John Kinsman

**Affiliations:** 1 European Centre for Disease Prevention and Control (ECDC), Stockholm, Sweden

**Keywords:** One Health, preparedness, cross-sectoral collaboration, policy, qualitative interviews

## Abstract

**BACKGROUND:**

One Health (OH) is a multisectoral approach that aims to optimise health of people, animals and the environment, recognising their interconnection. Despite gaining political support in recent years, examples of successful OH implementation among governmental institutions across the European Union/European Economic Area (EU/EEA) remain limited.

**AIM:**

To identify key enablers and barriers to collaboration across human, animal and environmental health sectors, and provide a framework to support national OH operationalisation in the EU/EEA.

**METHODS:**

Semi-structured interviews were conducted with 26 experts from national public health institutes in 15 EU/EEA countries, recruited through European Centre for Disease Prevention and Control networks. Transcripts were analysed through qualitative content analysis.

**RESULTS:**

Collaborations between human and animal health sectors were reported, but greater integration of the environmental sector is needed to strengthen OH partnerships. Analysis of opportunities and challenges highlighted key interlinked elements that can facilitate sustainable OH implementation. Strong political leadership emerged as pivotal to drive policy coherence in nexus areas, embed collaborative activities within core funding, and facilitate cross-sectoral partnerships at the technical level.

**CONCLUSION:**

This qualitative study provides an overview of enablers and barriers to OH collaboration at the national level. The findings constitute the basis for an empirically derived framework emphasising the cyclical relationship between political leadership and cross-sectoral technical collaboration. Incremental steps, starting with strengthening existing cross-sectoral relationships, have the potential to generate self-reinforcing progress and enhance emergency preparedness. These empirical insights provide a foundation for developing and evaluating OH policies in EU/EEA countries, complementing existing international guidelines.

**Key public health message**

**What did you want to address in this study and why?**

Collaboration across human, animal, and environmental health sectors — known as One Health — is critical to address shared health threats like emerging infectious diseases. While both scientists and politicians agree on its importance, implementing One Health within separate government structures is challenging. We wanted to understand the opportunities and challenges of implementing this approach at the national level in European Union/European Economic Area (EU/EEA) countries.

**What have we learnt from this study?**

We interviewed public health experts in 15 EU/EEA countries and found that successful collaboration depends on a combination of factors. Political leadership is essential to provide means for One Health action and set clear goals and responsibilities. Collaboration among experts that helps achieve positive outcomes can raise awareness and encourage further political action.

**What are the implications of your findings for public health?**

Small, strategic steps, such as strengthening existing partnerships through political support and targeted resources, can create a positive environment for implementing One Health. Our findings offer guidance for improving collaboration across sectors to better address complex health challenges and prepare for future emergencies.

## Introduction

One Health (OH) is an integrated approach that recognises that the health of humans, animals, plants and their shared environment is interconnected. It promotes collaboration across sectors – mainly of human, animal, and environmental health – to tackle shared health challenges at the animal-human-environmental interface and thereby to optimise the health of all [1]. It is estimated that over 60% of known infectious diseases and 75% of emerging infectious diseases in humans are zoonotic [2,3]. Ongoing anthropogenic change, such as climate change and biodiversity loss, further drives the risk of infectious disease emergence [4], underscoring a critical need for the OH approach. Beyond zoonotic diseases, antimicrobial resistance and environmental pollution present parallel global health crises requiring coordinated responses.

In recent years, political attention to OH has increased, especially following the COVID-19 pandemic, which highlighted the need for interdisciplinary approaches to address health threats at their source while ensuring social and environmental sustainability [5]. At the global scale, OH implementation has been championed by the Quadripartite collaboration (consisting of the World Health Organization (WHO), World Organisation for Animal Health (WOAH, founded as OIE), Food and Agriculture Organization of the United Nations (FAO), United Nations Environment Programme (UNEP)) through their Joint Plan of Action [6]. In the European Union (EU), OH has been endorsed by legal instruments such as Regulation (EU) 2022/2371 on serious cross-border threats to health [7] and the work of the EU Cross-agency OH Task Force [8].

Despite increasing political commitment to institutionalising OH, practical implementation of the approach remains complex [9]. Currently, operational examples of OH at the national level within the European Union/European Economic Area (EU/EEA) are limited, and success stories among competent authorities are not widely observed. The OH approach necessitates new ways of working, notably through strengthened intersectoral collaboration, driving the creation of new outputs. Moreover, ethical and legal tensions arising from conflicting values complicate decision-making processes [10]. This underscores the need for guidance to facilitate the operationalisation of OH, highlighting the importance of understanding the factors that make the approach actionable.

Challenges around OH implementation have been addressed in global policy documents, such as the OH Theory of Change [11]. In the academic literature, most publications studying OH implementation have focused on specific interdisciplinary projects [12] or non-European countries [13,14]. In the EU, some studies were conducted for example on the Swedish and Italian national contexts [15,16]. Repeatedly identified challenges include weak intersectoral coordination and communication, insufficient funding and a lack of political awareness.

However, the broader European context remains underexplored, leaving a critical knowledge gap regarding challenges and opportunities in national OH implementation. This study aims to address this gap by investigating the experiences of OH collaborations and visions for implementation at national level in EU/EEA countries through qualitative interviews. To our knowledge, this is the first qualitative study to provide an overview of enablers and barriers to OH operationalisation across competent authorities in this region. The findings contribute a novel empirical framework emphasising the cyclical interconnection of essential factors for successful OH implementation, providing a foundation for sustainable intersectoral collaboration, policy development and future research.

## Methods

### Study design

Data were gathered through semi-structured qualitative interviews with public health professionals working at national level in the EU/EEA.

### Recruitment of participants

**Figure 1:**
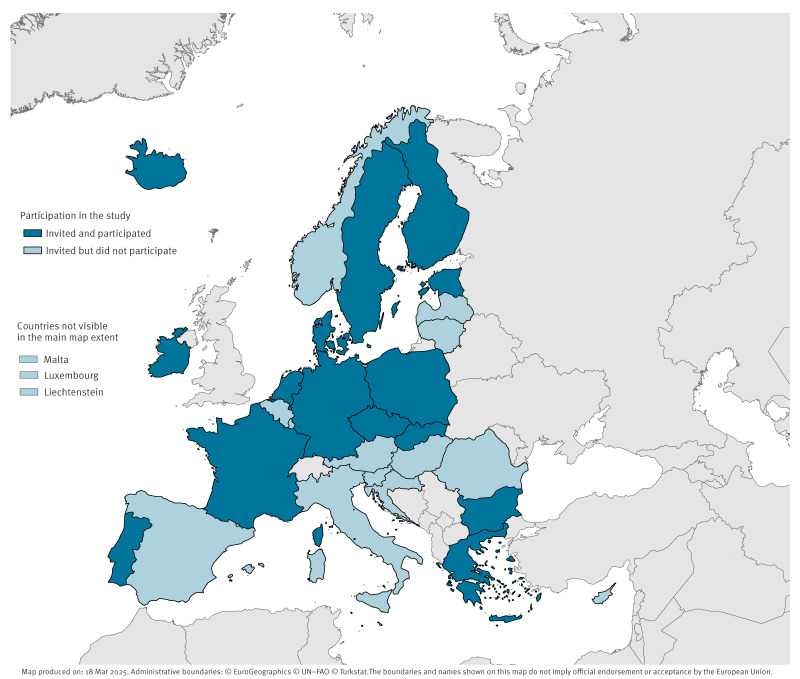
Countries with national public health institutions invited (n = 30) or participating (n = 15) in the study, EU/EEA, 2024

Potential participants were identified through convenience sampling, using European Centre for Disease Prevention and Control (ECDC) networks. The ECDC Coordinating Competent Bodies are designated institutions in each EU/EEA country that facilitate collaboration between ECDC and national authorities within the technical fields covered by ECDC’s mandate. Coordinating Competent Bodies from all 30 EU/EEA countries [17] were invited to nominate participants. Recruitment was carried out by the ECDC National Coordinators of Competent Bodies, who were approached by ECDC via email. National Coordinators were requested to nominate experts at national public health institutions with knowledge and experience in implementing the OH approach at the national level. Experts from 16 countries were nominated (from one to three experts per country) and after one drop-out due to personal circumstances, interviews were conducted – one per country – with a total of 26 active participants from 15 countries (multiple experts from the same country were interviewed jointly). All participants were contacted via email before the interview and provided with an information sheet outlining the study and a privacy statement for data processing. Figure 1 provides a map showing the countries associated with the public health institutions of participants.

EU/EEA: European Union/European Economic Area.

### Data collection

An interview guide was constructed comprising five guiding questions (see the Supplement for the complete interview guide), focused on identifying specific examples of OH collaborations at the national level. The interview guide was reviewed by one social science expert, two veterinary experts and one infectious diseases expert. The interview guide was sent to participants ahead of the interview, allowing time for consultation with colleagues as necessary. The interviews were conducted jointly by authors JH-B and CVS in August and September 2024. They were held online via Webex and were digitally recorded, lasting between 40 and 60 minutes. The conversations were semi-structured, and follow-up questions were adapted based on participants’ replies.

Verbal informed consent was obtained from the participants at the beginning of each interview. Participant anonymity was assured. 

Field notes were gathered during the interview and discussed by the interviewers shortly afterward, ensuring shared understanding of what was said and a consistent interpretation grounded in the context of the situation. Automatic verbatim transcripts generated by Webex were reviewed and corrected as necessary so that they corresponded to the recordings. Filler words were removed, and grammar was lightly edited to facilitate analysis and to reflect the accurate meaning of participants’ statements.

### Data analysis

Transcripts were subjected to qualitative content analysis, using Open Code software [18]. The questions in the interview provided an initial set of deductively derived themes, and these were progressively refined through an inductive process into sub-themes. The coding system was then jointly reviewed by authors JH-B, CVS and JK to eliminate overlaps and refine the analysis. Coded segments were reviewed to ensure their correct classification. A draft analysis of the data was sent to the participants for validation and feedback, which was incorporated into the final version. Quotations were included without attribution to preserve anonymity of participants. The Consolidated criteria for reporting of qualitative research (COREQ) guidelines [19] were used to provide a framework for reporting the findings of this study.

## Results

### One Health actors and collaboration structures

The first part of the interview aimed at obtaining an overview of the OH landscape in the country, focusing on actors and collaboration structures. A variety of institutions, across national and regional levels, were identified as OH actors. Although participants were not given a definition of OH before or during the interview, most of the actors they mentioned belonged to the sectors of human, animal or environmental health. Only a few participants noted the involvement of other sectors, such as ministries of the interior or foreign affairs, due to their role in health emergencies. A detailed list of the identified OH actors is presented in Table 1.

**Table 1:** One Health actors by level, category and number of countries reporting, EU/EEA, 2024 (n = 15 responding countries)

Level	Category	Actor	Number of countries reporting
National	Ministries	**Ministry of Health**	**14**
		**Ministry of Agriculture and Food**	**13**
		**Ministry of Environment**	**8**
		Ministry of the Interior	2
		Ministry of Foreign Affairs	1
		Ministry of Research	1
	Agencies	**Public Health**	**15**
		**Food safety, animal health, and agriculture**	**14**
		**Environment or plant protection**	**8**
		Medicines agency	3
		Institute for risk assessment	1
		Hydrometeorological institute	1
	Universities and national research institutes		6
	Reference laboratories for communicable diseases (human and/or veterinary)		5
	Non-governmental organisations in animal welfare and the environment		1
	World Health Organization country office		1
	Customs in ports and airports		1
Sub-national	Agencies (including subsidiaries of national agencies)	**Public Health**	**12**
		**Food safety, animal health and agriculture**	**9**
		Environment	3
	Hospitals or healthcare services		6
	Municipalities		5
	Diagnostic laboratories		2

EU/EEA: European Union/European Economic Area.

One health actors mentioned by more than half of the countries reporting are presented in **bold**.

When asked about underrepresented actors, multiple participants highlighted the need for greater participation from the environmental sector, which they felt was hindered by its unclear role and responsibilities in OH. Some participants identified additional actors who could play a more prominent role, such as the general public, occupational health authorities, private veterinary practices and animal production stakeholders.

Participants described varying levels of OH collaboration, defined from the perspective of national competent authorities, as two or more sectors working together on topics under the OH umbrella. These collaborations differed in scope and timeframe. Participants from nearly half of the countries reported having a strategic OH coordination group involving high-level representatives from public, animal and environmental health sectors. Participants from all countries reported structural collaboration at the technical level, typically through regular intersectoral meetings on specific topics. The most frequently mentioned areas of collaboration were food-and waterborne diseases and antimicrobial resistance (AMR). A complete overview of reported collaboration types is outlined in Table 2.

**Table 2:** Types of One Health collaborations, EU/EEA, 2024 (n = 15 responding countries)

Collaboration type	Objectives	Actors	Nature of collaboration	Number of countries reporting
OH coordination groups	Overarching, strategic focus to coordinate actions in OH	High-level representatives from competent authorities in public health, animal health and environmental health	Structural activity	7
Technical groups	Regular, topic-specific meetings at technical level, e.g. on food- and waterborne diseases, AMR, zoonoses, avian influenza, vector-borne diseases	Technical-level representatives from competent authorities	Structural activity	15
Informal technical networks	Information and data exchanges, relying on personal relationships between counterparts	Technical experts from competent authorities	Ad hoc	11
Collaboration between competent authorities and academia	Joint research projects or ad hoc collaborations during health emergencies	Academic researchers and technical experts from competent authorities	Project-based or ad hoc	8
International projects	Topic-specific collaboration for data exchange or joint research projects, e.g. OH SURVector [27], UNITED4Surveillance [28], EU JAMRAI [29], the European OH Association [30]	Technical experts from competent authorities from several countries and sometimes academia	Project-based	6
Sub-national level collaboration	Technical collaboration at sub-national level: topic-specific or ad hoc during health emergencies	Technical experts from regional competent authorities, local human and animal health inspectors	Structural activity or ad hoc	4

AMR: Antimicrobial Resistance; EU/EEA: European Union/European Economic Area; EU JAMRAI: European Joint Action on Antimicrobial Resistance and Healthcare-Associated Infections; OH: One Health.

### Thematic analysis of enablers and barriers to One Health implementation

The analysis of the remaining interview data identified key enablers and barriers to collaboration. These were grouped into a set of inductively derived thematic categories, which are detailed below. An overview of the main enablers and barriers is presented in Table 3.

**Table 3:** Overview of the main enablers and barriers by category, EU/EEA, 2024 (n = 26 participants)

Category	Issue	Enabler	Barrier
Political leadership	Hierarchy at all levels is aware of OH and supports collaboration	X	
	High-level coordination body for intersectoral leadership	X	
	Common interest of sectors, e.g. win-win objectives	X	
	Lack of political awareness and endorsement of OH		X
	No common vision, weak leadership or coordination		X
	Conflicting interests between sectors, e.g. public health vs agricultural livelihoods		X
Policies and legislation	National strategies promoting collaboration, e.g. AMR action plans	X	
	Legal obligation to act jointly or share information, e.g. food-borne outbreak response	X	
	Clear definition of actor’s roles and responsibilities	X	
	Separate sectoral legislation, unregulated nexus areas^a^ and legislative grey zones		X
	Restrictive data sharing regulations		X
Funding	Sustainable, dedicated resources for routine OH activities including joint tools and regular meetings	X	
	Funding for international OH projects	X	
	Fragmented sectoral budgets with no specific allocation for collaboration		X
	Funding for collaborative activities relies only on projects, disrupting continuity when resources tighten		X
	Resource disparities between sectors and competition for same funds		X
Technical cross-sectoral collaboration	Joint working methodologies, tools and platforms, e.g. data sharing, emergency management	X	
	Regular cross-sectoral expert meetings and opinion exchanges, awareness of counterparts’ activities	X	
	Personal intersectoral networks and engaged experts	X	
	Standardised communication pathways relying on roles	X	
	Lack of common terminology and methodology, e.g. risk assessments		X
	Poor interpersonal communication, lack of training in collaboration and networking		X
	No formalised communication pathways, staff turnover breaks links		X
Advocacy	Requests to governments by EU and international organisations; expert groups highlighting policy gaps	X	
Health emergencies	Acute events acting as triggers to align sectors around a shared, time-bound purpose	X	

AMR: antimicrobial resistance; EU: European Union; EU/EEA: European Union/European Economic Area; OH: One Health.

^a^ Areas that are inherently overlapping between sectors.

### Political leadership

Political leadership was identified as a key enabling cornerstone of OH at the national level. Awareness and endorsement of the OH approach by political actors at all levels, including ministers, agency directors and local mayors, is pivotal to fostering collaboration. Conversely, a lack of political awareness and endorsement of OH, and its absence from political negotiations, was mentioned as a notable barrier to collaboration. Understanding of the OH approach should also extend to institutions not primarily active in health, but involved in health emergencies, such as ministries of the interior, agriculture or others.

Multiple participants reported that the political level should not only endorse the OH approach but also provide leadership and coordination. Firstly, the governmental level can lead legislative change and provide incentives, both of which may be necessary to drive implementation. Collaborative structures can benefit from the presence and endorsement of political and managerial levels to obtain means to action. Secondly, the OH approach requires a long-term political vision, due to the breadth of the concept and the difficulty in reaching a shared, actionable strategy across sectors. Siloed interests of involved sectors play a notable role: when their objectives align, collaboration is facilitated, potentially leading to win-win outcomes. However, when objectives diverge, competition for power and conflicting interests between sectors were cited as key barriers:

“*The interests of public health and animal health could clash because [in the animal sector] you have sometimes just the interest to raise animals and to sell them in the end, which is not exactly the same as public health.*”

To achieve a high-level political vision guiding competent authorities towards common goals, several participants mentioned the benefits of OH governance structures, such as a high-level coordination body.

Led by government officials from relevant sectors, such structures give authority to the approach. Unlike collaborative committees at the technical level, a high-level body can be pathogen- and project-independent, allowing for negotiation of a broad political vision and provision of overarching coordination. It can be an effective platform for aligning interests, messaging and methodologies between sectors, while also serving as the starting point for implementing joint OH strategies.

### Policies and legislation

Driven by political leadership, the design and wording of policies and legislation can either promote or hinder OH collaborations. Policies that encourage the OH approach, such as a national AMR strategy with associated collaborative goals, were cited as facilitating factors to collaboration at the technical level. Additionally, some participants mentioned that certain legal obligations in their country, such as the requirement for public health and food safety sectors to exchange information on food-borne outbreaks, can enable collaboration if partners choose to conduct joint investigations.

Conversely, multiple participants reported challenges with sector-specific legislation that does not regulate nexus areas — areas that are inherently overlapping between sectors — causing friction. Data sharing laws were frequently cited as particularly restrictive, leading to fragmented surveillance and challenges in establishing routine data exchange between sectors. Importantly, collaboration functions better when roles and responsibilities of competent authorities are clearly defined and distributed, which in many countries is best achieved through supportive legislation. Unclear roles and responsibilities can hamper work in nexus areas, leading to fragmentation, power struggles, or inertia:

“*Sometimes the authorities overlap, and all may do the same thing, and sometimes no one does anything.*”

Finally, policies and laws that clearly mandate the use of a OH approach provide a basis for accessing resources to build, improve and sustain collaborative structures.

### Funding

Funding priorities, set by the political leadership, shape the extent to which OH initiatives are possible. Funding can enable collaborations through joint projects, and it can also facilitate routine activities of competent authorities. Joint international projects offer opportunities to exchange best practices among countries and can serve as pilots to gain OH experience at the national level. In routine activities, funding was regarded as essential to develop collaborative tools and implement joint activities such as integrated surveillance. Some participants highlighted the importance of ‘buying’ people’s time, for example to organise regular meetings, which are necessary for building trust among collaborators:

“*Time is money, but money is time too.*”

An important barrier is the shortage of resources for initiating and sustaining collaborations: OH activities are often assumed to be covered by core budgets, when in fact participants reported that they require additional resources and time. Moreover, funding is typically fragmented across sectors, complicating the financing of initiatives in nexus areas that need joint funding efforts. Sectors may also sometimes compete for funds, struggling to secure resources for their own priorities. Furthermore, there may also be substantial resource disparities between institutions, with the environmental sector often being underfunded for health-related activities.

Many OH activities rely on project-specific funding, with resources for cross-sectoral collaboration rarely prioritised and included in the core budgets of competent authorities. This jeopardises the continuity of collaborative activities when resources are limited. This structural issue is particularly pronounced in small countries with limited public health workforce that are struggling with competing priorities, which further threatens the sustainability of OH collaborations:

“*There is always a concern that the network formed or strengthened through a funded initiative will die away.*”

### Technical cross-sectoral collaboration

Effective OH collaboration at the technical level requires establishing tools, methodologies and interdisciplinary dialogue. Participants reported that joint working tools, including platforms for data sharing and emergency management, facilitate collaboration. Conversely, some participants highlighted the considerable challenge posed by the lack of common methodologies for data collection and risk assessments across sectors, which can prevent data comparison and joint conclusions. Therefore, cross-sectoral collaboration necessitates sustained communication between experts to exchange opinions and analyses, align on situation awareness, and develop joint methodologies.

Personal relationships, through experts knowing their counterparts in other sectors, were seen as essential by many participants, as they favour collaboration and timely informal exchange:

“*In case we need cooperation usually we know who to call personally and to establish the measures needed to solve the problem*.”

These networks are often naturally established in small countries with a limited OH workforce and a high level of awareness of counterparts’ roles. Interpersonal skills are crucial for maintaining strong networks: some participants cited difficult interpersonal relationships as a barrier to collaboration at the technical level. Engaged experts, who understand the value of collaboration and who have the scientific curiosity to build bridges to solve interdisciplinary questions, are key. This engagement can be promoted by managers through rewarding collaborative approaches over competition, favouring knowledge exchange (including with experts in other countries), and hiring staff with strong networking skills.

Although personal intersectoral relationships are essential for effective collaborations, relying solely on informal networks without establishing clear procedures for intersectoral work is unsustainable, as there is no continuity when personnel change their roles within organisations. Therefore, clear communication pathways relying on formalised interaction procedures and standardised processes were regarded as enablers to OH collaboration. Regular contact and cross-sectoral expert meetings enable timely resolution of issues, ensure transparency, and maintain connection between organisations. Simulation exercises can provide a platform to test and reinforce these interaction procedures. By relying on formalised processes, these communication pathways ensure the longevity of cross-sectoral collaborations:

“*It's really important not just to have the name of somebody you can call but [also] the role; that's what makes it [the collaboration] last*.”

### Advocacy

Effective technical collaboration can achieve success stories that facilitate advocacy efforts, thereby enhancing awareness among political leaders and promoting supportive policymaking. Established cross-sectoral expert groups that identify legislative grey areas and policy gaps can serve as effective advocacy platforms and propose supportive strategies for collaboration. Some participants highlighted the importance of training natural leaders who may become OH champions, advocating for the cause at both political and technical levels. Lastly, the EU and international organisations can play a pivotal role in OH advocacy at the national level, by showcasing benefits of OH work, and issuing requests that trigger cross-sectoral collaboration:

“*If the ministries get pressure from the EU or if whatever international organization says, ‘you should do this’ or ‘we need this’, then they are more likely to actually put it on the agenda*.”

### Health emergencies

Health emergencies, such as outbreaks of zoonotic influenza, vector- or food- and waterborne diseases, were frequently cited as triggers for OH collaborations. These events create a common purpose fostering collaboration among experts, who may then practice cross-sectoral communication through joint operational work during emergencies and thereby establish personal relationships with their counterparts. Shared success stories can strengthen these bonds and enhance OH advocacy at both political and technical levels, potentially leading to changes in guidance. This may result in durable cross-sectoral collaborations that continue post-emergency:

“*People get to know each other and then they can work together in future emergencies, and this improves preparedness*.”

Urgency and public attention also boost the enabling process at the political level:

“*You cannot communicate to the public that you did not cooperate in an outbreak*.”

## Discussion

Our study identified numerous national level collaborations between human and animal health sectors, aligning with previous findings in the European region [20]. However, consistent with earlier observations [11,16], the environmental sector remains underrepresented and requires greater integration into OH initiatives. Previous findings suggest that this limited involvement may reflect the environmental sector’s focus on priorities other than OH [16]. Our results further indicate that this gap could also result from unclear delineation of potential roles and responsibilities in OH, and by underfunding of environmental agencies for health-related activities. Recognising these barriers, the Quadripartite’s OH Joint Plan of Action [6] prioritises the integration of the environmental sector through key measures, including: (i) advocating for the recognition of the critical role of environmental health in human and animal health and wellbeing, (ii) integrating environmental stakeholders into national OH strategies, and (iii) developing OH in-service training programmes including environmental health topics for all sectors. Collectively, these actions have the potential to provide the environmental sector with more resources for engagement in OH.

By analysing enablers and barriers to collaboration at national level in the EU/EEA, our study identified several structural challenges to OH implementation. These include policy incoherence, resource constraints and limited political commitment, resonating with previous findings, including those from other geographical regions [13,14,16].

**Figure 2:**
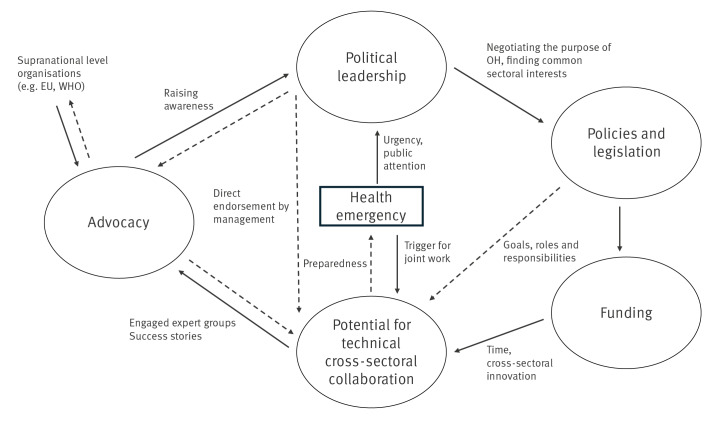
Framework for enabling sustainable One Health collaboration at national level in the EU/EEA

EU: European Union; EU/EEA: European Union/European Economic Area; OH: One Health; WHO: World Health Organization.

Based on the links between the inductive categories identified in our study, we propose a framework that can be used to facilitate both scientific understanding and practical advancement of OH collaborations in the EU/EEA. This empirical framework validates the interconnection between key elements of OH governance, conceptualised in systems thinking approaches and evaluation tools [21,22]. These elements have also been recognised in major OH guidance documents from international organisations [6,23] and in the recently published Lancet OH Commission report, recommending strengthened OH governance though coherent policy-making and equitable financing [24,25]. The interlinked, cyclical relationships between the framework’s categories are shown graphically in Figure 2.

Solid arrows indicate main, cyclical relationships between categories, derived from the results of the study. Dashed arrows indicate other possible links between categories.

A key insight from this framework is the importance of strong political leadership for OH. Implementation of the approach is not solely a technical challenge: it requires high-level political leadership [9,11] to establish a long-term vision, negotiate trade-offs and define actionable strategies with clear objectives. Incremental steps, such as strengthening existing OH networks through targeted resources and recognition, can generate considerable ripple effects. Policy coherence is key for effective OH governance. Harmonising policies across sectors clarifies roles and responsibilities in nexus areas and facilitates sustainable funding. Dedicated resources for technical collaboration are essential, and not only at the initial stages of implementation, to strengthen intersectoral relationships, develop and improve joint working tools, and enable collaborative activities. Regular interaction allows sectors using different terminology, e.g. for risk assessment, to reach common understanding and establish shared methodologies for joint work. This can be particularly challenging for small or under-resourced countries with limited public health workforce that must focus on core tasks and struggle with competing priorities. To ensure sustainability of OH activities, it is essential that trans-sectoral resources for collaboration become integrated into structural budgets rather than solely relying on project funding.

Intensified OH collaborations at the technical level not only foster learning and innovation [11] but also create advocacy opportunities. Success stories emerging from cross-sectoral efforts amplify political awareness and support for OH, facilitating the possibility for perpetuating a sustainable enabling environment.

Health emergencies can trigger this enabling cycle, as they demand coordinated efforts and highlight the value of integrated approaches. The framework supports the idea that health emergencies can drive advocacy and political support for OH, consistent with research showing that crises can initiate a virtuous cycle, leveraging OH literacy to improve governance [26]. However, solely relying on crises as enablers is not sustainable. Our framework emphasises the importance of bottom-up advocacy by established groups and policy entrepreneurs, that promote OH issues from the technical level to the political agenda [16], to strengthen OH governance.

All in all, the cyclical reinforcement between political leadership and technical collaboration offers a pathway to sustainable OH governance. Addressing structural barriers, such as competing sectoral interests, requires a shared political vision and dedicated, long-term funding. Strengthening and empowering existing networks is key to sustaining collaboration and enhancing preparedness for future crises.

Some limitations to our work should be acknowledged. Selection of participants was based on a convenience sample. While our participants were chosen for their experience in OH in their country, most were from the Public Health sector. Responses to our call for interview participants came proportionately more from western and northern EU/EEA countries than from central and southern countries. The framework could be developed further by including perspectives from other OH sectors, such as animal and environmental health. Furthermore, we acknowledge that participant’s views reflect individual opinions, not necessarily those of their organisations or countries.

## Conclusion

This study identified enablers and barriers to OH collaboration at the national level in the EU/EEA, and it provides a framework linking key elements facilitating sustainable OH implementation. Incremental action, starting with strengthening existing cross-sectoral collaborations, can create self-reinforcing progress. Strong political leadership is essential to drive implementation and ensure long-term commitment, building resilient OH systems capable of addressing current and future health challenges. Our findings offer empirical insights to complement national and international guidelines, serving as a foundation for OH policymaking and evaluation across EU/EEA countries.

Supplement

## Data Availability

Anonymised data that support the findings of this study are available from the corresponding author JH-B upon reasonable request.

## Supplementary Data

381000
