# Supplementary material for: A framework for facilitating sustainable One Health collaboration across sectors at the national level in the European Union/European Economic Area
Source: Euro Surveill. 2025 Oct 30;30(43):2500218. doi: 10.2807/1560-7917.ES.2025.30.43.2500218 (PMC12579316; doi:10.2807/1560-7917.ES.2025.30.43.2500218)
Supplement: Supplementary file 1 [file 25-00218_HOEFLE-BENARD_Supplement.pdf]

Supplementary material: interview guide

Introduction:

- Introductions of interviewers and interviewee(s)
- Introduction to the project (rationale and purpose)
- Explanation of the process and confidentiality policy

1. Which are the main One Health (OH) actors in your country? *(No need for a comprehensive list, just to understand the institutional structures)*

- Please describe their respective OH roles and responsibilities, and the level/s (i.e. national, regional, local etc.) at which they work.
- Are there any other actors who you think should be engaged but who are not engaged? If yes, why is this?

2. To what extent and how do those actors collaborate? *(No need for a comprehensive list, just to understand the institutional structures)*

- Who works with whom? On what topics?
- Are these collaborations predominantly formal (i.e. legal/policy basis) or informal?
- How is information shared between sectors? How are collaborations initiated and maintained? Please give an example.

3. Please give an example of what you would consider to be a successful OH collaboration in your country

- Who are/were the actors involved?
- What is/was the topic?
- Why do you think it is/was successful?
- What did/has it achieve(d)?
- Is it ongoing work or has it ended? Please explain why.

4. Please give an example of where you think that a OH collaboration did not work so well in your country

- Who are/were the actors involved?
- What is/was the topic?
- Why do you think it is/was unsuccessful? What are/were the challenges? Are those challenges you face on a regular basis? Do you think these challenges are resolvable and if so, how could they be resolved?
- What was the follow-up? Please explain.

5. What is your vision for OH in your country?

- What would you need to make this happen? (including how to address any challenges you foresee)
- How could ECDC/other EU agencies support you in accomplishing your vision?

Closing:

Do you have any questions or is there anything else you would like to add?

Thank you for your participation in this interview.
